# Exploiting synergies between microbial electrochemical technologies and synthetic biology

**DOI:** 10.1111/1751-7915.14208

**Published:** 2023-01-09

**Authors:** Bin Lai, Jens Krömer, Federico Aulenta, Hui Wu, Pablo Ivan Nikel

**Affiliations:** ^1^ BMBF Junior Research Group Biophotovoltaics Helmholtz Center for Environmental Research – UFZ Leipzig Germany; ^2^ Systems Biotechnology Group Helmholtz Center for Environmental Research – UFZ Leipzig Germany; ^3^ Water Research Institute (IRSA) National Research Council (CNR) Rome Italy; ^4^ State Key Laboratory of Bioreactor Engineering, School of Biotechnology East China University of Science and Technology Shanghai China; ^5^ The Novo Nordisk Foundation Center for Biosustainability Technical University of Denmark – Kgs Lyngby Denmark

## INTRODUCTION

Biotechnology and bioeconomy are the main driving forces steering the transition towards a more sustainable future. A variety of approaches to avoid, reduce and remove CO_2_ emissions will be essential to achieve net‐zero carbon emissions by 2050 as called for in the Paris agreement (Mengis et al., 2022). Over the past few years, synthetic biology has become a consolidated alternative towards creating and implementing pathways for the biosynthesis of relevant chemicals and optimizing microbial growth and production in biotechnological processes (Calero & Nikel, 2019; Tang et al., 2021). Since most biotechnological processes are not yet competitive compared with traditional chemical approaches used for synthesis, improving the yield, productivity and titre is critical for the economic success of bioproduction processes.

An intrinsic metabolic constraint for the yield is the mass and redox balance between substrate(s) and products, which determines the theoretical upper boundary of fermentations and accordingly the often‐inevitable formation of undesired by‐product(s), whose production often derives from the need of recycling of electron carries to balance the redox reactions involved in the cellular metabolism (Flynn et al., 2010).

To tackle this issue, microbial electrochemical technologies (MET) provide a unique opportunity to overcome this metabolic constraint (Schievano et al., 2016). In contrast to classical biological processes, MET balances the cellular electron flux using, virtually inexhaustible, external electrodes. The (partial) decoupling of the carbon and electron balance for the host microorganism can thus push the bioproduction process to a new upper boundary (Kracke & Krömer, 2014; Lai et al., 2016).

Synthetic biology also offers great opportunities for MET (TerAvest & Ajo‐Franklin, 2016). The performance of model electrogens could be engineered and improved using synthetic biology approaches (Tan et al., 2016). Non‐native electrogens, especially industrially‐relevant microbial hosts (Liu et al., 2017; Schmitz et al., 2015), become of increasing interest in MET by, e.g. introducing an extracellular electron transfer (EET) pathway. Moreover, the implementation of MET in real‐life applications can also be exploited, as illustrated by, e.g., a modular‐designed MET‐based biosensor for pollutant detection in aqueous environments (Atkinson et al., 2022a).

## MOLECULAR UNDERSTANDING OF EET PATHWAYS

Genetic engineering has been used to reveal the molecular mechanisms of EET pathways of model electrogens over the past decades. For instance, the redox proteins involved in the MtrCAB pathways of *Shewanella* to respire on minerals were studied by a series of mutation studies on each component. Such a full understanding of the EET pathways could pave the way to transplant them into different non‐native electrogens. In 2011, the EET pathway was found to be able to run in reverse (Nevin et al., 2011), i.e., feeding electrons from the cathode into the microbial redox metabolism. The inward EET flux from the electrode to the cells forms the basis for the discipline called *microbial electrosynthesis*, especially for reducing CO_2_ into value‐added chemicals via (bio‐) electrochemical reduction process. The molecular mechanisms for the inward electron transfer pathway have been the subject of intense debate for quite some years (Rosenbaum et al., 2011). One such argument revolved around the essentiality of H_2_ to mediate this cathodic EET, as both cases (with and without H_2_) are supported by some experimental evidence. The participation of H_2_ in the EET seems to be strain‐ and environmental condition‐dependent. In this special issue, (Tefft et al., 2022) took a step further to study the inward EET pathway of *Shewanella* at the molecular level by creating a series of deletion mutants. In doing so, the authors found that the activity of the inward EET pathway is also dependent on the electron sinks from production pathways. NADH dehydrogenase was essential to deliver electrons from cathode to NADH‐dependent pathways, e.g. from acetoin to 2,3‐butanediol—but it was not needed if fumarate was present as the electron acceptor. These results indicate a highly dynamic inward EET pathway, which could be tailored for specific purposes by considering the strain, system configuration and microbial physiology.

## ELECTRONIC CONTROL OF GENE EXPRESSION

Controllable gene expression is a major goal when introducing new pathways or directing metabolic flux to new products in synthetic biology and metabolic engineering. While many processes are controlled by the addition of (often, one‐time‐use) inducers, the respective costs can increase quite significantly—especially when considering demands at an industrial scale. Using cheap, renewable electricity to induce gene expression is a good alternative to enhance the economic feasibility of a biosynthetic process. In 2017, electronic control of gene expression was reported in *Escherichia coli* (Tschirhart et al., 2017). Electric inputs were transformed into biological responses via redox carriers. Ferricyanide lifted the redox potential of pyocyanin to an oxidative status that triggered gene expression, and the resulting ferrocyanide was recycled back to ferricyanide at the anode. A continuous and controllable electronic gene expression system was thus established, which can be implemented in different biological systems.

Tunable (or even self‐tuning) gene expression is also desired at different process phases. A genetic toggle switch was reported in 2000 (Gardner et al., 2000), presenting a smart concept of self‐controlled target gene expression and phenotype shift by two repressible promoters. The cell function can be tuned between two states upon the interactive activation and repression of the two promoters by their respective inducers. To integrate this toggle switch with electronic gene expression control, (Grozinger et al., 2022) present a mathematical model for the electrogenetic toggle switch design within this special issue. They addressed the bistable region and how it would be affected by different running parameters in a homogenous model and by spatial heterogeneity. Although further experimental validation is needed, this analysis provides a first‐step theoretical guideline for the future design of biological electrogenic toggle switch to rationally change the microbial behaviours.

## NOVEL MICROBIAL HOSTS FOR MET

*Shewanella* and *Geobacter* are used as model organisms for MET studies, owing to their naturally evolved ability of using minerals as respiratory electron acceptors. However, the first documented work on bioelectric output was reported in the yeast *Saccharomyces cerevisiae* (Potter, 1911). Fungal MET research was recently reviewed (Sarma et al., 2021). So far, these MET systems were mostly applied for wastewater treatment, but they hold strong potential for bioproduction purposes, as many fungal species are relevant for industrial biotechnology. Rationally tuning phenotypes to match the electrochemical environment remains a major challenge, which requires intensive efforts on strain engineering. In this special issue, (Wang, et al., 2022a) comprehensively reviewed the development of CRISPR‐Cas tools for the fungus *Trichoderma*. Different transformation methods and strategies to introduce Cas nucleases and guide RNAs into the fungal cells are compared in this article, as well as the potential application scenarios and future perspectives.

The microbial host spectrum in MET research was greatly expanded by the mediate‐based EET pathway. One representative example was the discovery and application of the natural phenazine and its derivatives, which has been demonstrated to mediate the interaction between electrodes and several non‐native electrogens, e.g., *Pseudomonas putida* (Schmitz et al., 2015), *E. coli* (Feng et al., 2018), etc. The molecular mechanism of this phenazine‐based EET and its efficiency remain to be further clarified and improved, which, however, could likely be strain and environmental conditions‐specific. In this special issue, (Franco et al., 2022) screened over 100 seemly phenazine producers based on their genomes. A strong mismatch between the genome capability and the active phenotype was found, and only five species were finally demonstrated on the physiological level with reasonable EET activities. This work reported several new microbial hosts that can potentially be used for further electro‐fermentation research. Moreover, it also pointed out the importance of strain selection and microbial physiology that have to be considered while utilizing the self‐secreted mediator in MET for further bioproduction purposes. A systematic and comparative analysis and balance of the mediator synthesis pathway with other metabolic activities (e.g., the biosynthesis of target product(s), etc.,) will be essential.

The characteristics and potential of another non‐model MET microorganism for MET research, *Marinobacter*, were also reviewed by Bird et al. (2022) in this special issue. To this end, the authors discussed the strengths and limitations of *Marinobacter* for synthetic MET applications from the metabolic and electrochemical viewpoints. *Marinobacter* exhibits some unique features that can be beneficial for MET applications. For instance, some strains have strong biofilm formation capabilities on different solid surfaces (e.g., carbon, glass and metal), which is vital for direct EET. Other members of the genus also show high resistance to environmental stress and harbour, e.g., halophilic pathways and/or lipid production pathways, making them great candidates for electrobiosynthesis of chemicals that are typically toxic to other microorganisms. However, like many other non‐native electrogens, there are big knowledge gaps on how *Marinobacter* can interact with the electrode and the application potentials determined by, e.g., the EET rate—among other open questions. Genetic engineering tools should also be developed to tailor *Marinobacter* genotypes in the MET system for the desired purpose.

Among non‐model electroactive microorganisms, purple phototrophic bacteria (PPB) are also recently attracting considerable attention for their photoelectroautotrophic metabolism, which holds promise in the broad context of carbon fixation. Along this line, Manchon and colleagues (Manchon et al., 2022) explored for the first time the cultivation of a PPB‐dominated consortium and reported the occurrence of biomass growth with a polarized (−0.6 V vs. Ag/AgCl) cathode serving as the electron donor. The application of electrochemical techniques also provided preliminary indications regarding the involved EET mechanism, which still appears to be a major rate‐limiting step in the process development and future exploitation.

## EMERGING APPLICATIONS OF MET

Microbial electrochemical technologies have experienced almost two decades of exponential growth, but only few processes were or are being exploited at the industrial level, e.g., the Aquacycl (USA) and iMETland (Spain) technologies. Bridging the ‘valley of death’ between lab‐based processes and industrial applications is critical for the continuous growth of MET.

Most MET studies are based on biofilm platforms. The electrochemical activity of biofilm intrinsically determines the overall turnover rate—thus, the upper process boundaries for potential application scenarios. One key problem is decreasing biofilm activity with increasing biofilm thickness, mainly due to the mass and electron transfer limits within the biofilm. In this special issue, (Wang, et al., 2022b) reported on their efforts to tackle this issue (at least to a certain degree) by using a synthetic biology approach. The authors overexpressed several nanowire subunits of *Geobacter sulfurreducens* and they found that the mutants could form 45% to 70% thicker biofilms. Higher total power outputs were achieved with these engineered strains as compared to the wild‐type, and the specific power output normalized to the biomass content was likewise enhanced. How long this positive correlation between biofilm thickness and specific biofilm activity could last (overall turnover rate and productivity of the MET‐based process) will be the subject of future research.

The article indicated above reports a clear example of how synthetic biology can help to tackle the current challenges towards the broad application of MET. Similar concepts can be adapted to other scenarios. To realize all these potentials, a deeper understanding of the MET process will be critical. In this special issue, (Atkinson, et al., 2022b) presented a systematic, comprehensive and also introductory review for synthetic bioelectronic design. The MET process components were categorized by the authors based on their functions and behaviour, similarly to the classic circuit engineering category. The state‐of‐art is presented for each component in the context of its relevance and potential for bioelectronic device design. This review discusses the feasibility of a novel modular design approach to assemble different bioelectronic components for multiple purposes—with synthetic biology as a main enabling technology.

In summary, several examples in this special issue underscore the synergies between MET and synthetic biology. Both fields will gain unique new opportunities and potentials by integrating with each other. Quantitative understanding and the subsequent rational design will be challenging—but they also constitute the basis for future developments at different levels of implementation.

## Author contributions

Bin Lai drafted the manuscript. Jens Krömer, Federico Aulenta, Hui Wu and Pablo Ivan Nikel reviewed and edited the draft. All authors read and approved the final paper.

## CONFLICT OF INTEREST

The authors declare no conflict of interest regarding the content of this editorial.
